# A complicated case of refractory multiple drug-resistant peritoneal dialysis-associated peritonitis due to teratoma

**DOI:** 10.1016/j.idcr.2025.e02209

**Published:** 2025-03-31

**Authors:** Li-Yan Mao, Yi Tang, Juan Yang

**Affiliations:** a Department of Laboratory Medicine, Tongji Hospital, Tongji Medical College, Huazhong University of Science and Technology, Wuhan 430030, China; b Tongji Medical College, Huazhong University of Science and Technology, Wuhan 430030, China; c Department of Nephrology, Tongji Hospital, Tongji Medical College, Huazhong University of Science and Technology, Wuhan 430030, China

**Keywords:** Peritoneal dialysis, Peritonitis, Teratoma, Multidrug resistance

## Abstract

Peritoneal dialysis-associated peritonitis is a leading cause of treatment discontinuation and mortality among peritoneal dialysis patients. The presence of multidrug-resistant organisms further complicates management, particularly in patients with complex intra-abdominal conditions. This paper presents a complicated case of refractory multidrug-resistant peritoneal dialysis-associated peritonitis, which was ultimately diagnosed as being caused by a teratoma. Following adequate drainage and the administration of sensitive antibiotics, the patient successfully underwent teratoma excision and transitioned to hemodialysis.

## Introduction

Peritoneal dialysis (PD) is a well-established renal replacement therapy for patients with end-stage renal disease (ESRD), peritoneal dialysis-associated peritonitis (PDAP) is a leading cause of hospitalization, treatment failure, and mortality in PD patients, often necessitating a switch to hemodialysis or, in severe cases, resulting in death. While standard antibiotic therapy can resolve most cases of PDAP, but the infections caused by multidrug resistance (MDR) organisms are particularly concerning due to their severe limitation of treatment options and correlation with elevated treatment failure, morbidity, and mortality [1].

Teratomas, especially mature cystic teratomas, are rare germ cell tumors that may complicate if large or infected [2]. When associated with MDR peritonitis, the combination of infection and tumor mass complicates diagnosis and treatment [3]. This case report details an exceptional instance of refractory MDR peritoneal dialysis-associated peritonitis in a patient with an abdominal teratoma, underscoring diagnostic and therapeutic complexities and the necessity of a multidisciplinary approach.

## Case presentation

A 36-year-old female patient was admitted on October 16, 2023, with a history of maintenance peritoneal dialysis for 11 years. She presented with intermittent fever and abdominal pain for over a month. Eleven years earlier, she began PD due to renal insufficiency. One month prior to admission, she experienced persistent abdominal pain and fever without identifiable triggers. The pain was localized to the umbilical and lower abdomen, described as intermittent dull pain, accompanied by cloudy ascites. Fever predominantly occurred in the afternoons and evenings, reaching a maximum of 39.2°C, with blood pressure fluctuating between 80 and 90/50–60 mmHg.

Initially, the patient underwent standard anti-infective therapy at an external facility but showed an inadequate response. Antibiotics were escalated, yet the patient remained febrile. Microbiological cultures of ascites identified *Candida fermentati*, prompting the initiation of fluconazole; however, the fever persisted. On September 13th, the patient underwent laparoscopic removal of the peritoneal dialysis catheter under general anesthesia, during which an intra-abdominal abscess was identified and drained. Culture from the catheter tip again confirmed *Candida fermentati*, leading to the initiation of caspofungin therapy. Despite this, the patient’s fever continued. Subsequent cultures drainage fluid from abdominal abscess revealed infection with extensively drug-resistant *Pseudomonas aeruginosa*, with susceptibility to polymyxin, but therapy was halted due to neurotoxicity. In addition, the patient's microbial culture results also showed the presence of other *Gram-positive* and *Gram-negative* bacteria, Supplemental Fig. 1 showed the details of microbial culture results and the treatment course. Although blood pressure improved during hospitalization, intermittent fevers persisted, leading to a diagnosis of PDAP. The patient's medical history included an arteriovenous fistula creation in the left arm in 2018, which later occluded, and an inguinal hernia repair (date unknown).

Initial Computed Tomography (CT) scan at the external hospital (September 2023) revealed a mixed-density mass measuring approximately 9.3 cm × 7.8 cm in the lower abdomen and pelvis, containing calcifications and fatty components. Vital signs on admission included a temperature of 36.3 °C, pulse of 80 beats per minute, respiratory rate of 18 breaths per minute, and blood pressure of 141/100 mmHg. The patient appeared malnourished and anemic, with a palpable mass in the lower abdomen.

**Fig. 1:**
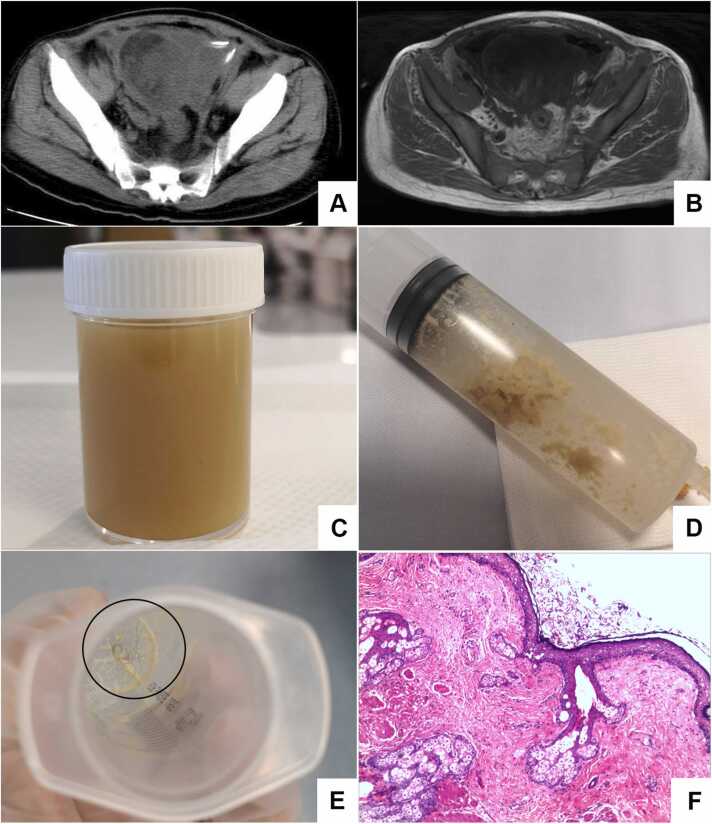
Images from the patient’s diagnostic and treatment process. A) Non-contrast Computed Tomography (CT) scan of the whole abdomen (including upper abdomen, lower abdomen, and pelvis) with tomographic imaging.; B) Magnetic Resonance Imaging (MRI) scan of the pelvis without contrast (including diffusion-weighted imaging); C) Drainage of abdominal abscess fluid; D) Fat-like substance flushed from the abdominal cavity; E) Hair-like substance flushed from the abdominal cavity; F) Pathological findings of the abdominal mass post-surgery: H&E stain, 200 × magnification.

On admission, laboratory tests revealed an elevated Leukocyte (17.97 × 10^9^/L) with neutrophilia (15.17 × 10⁹/L), increased white blood cell (WBC) count of PD fluid (2000 cells/mm³, predominantly neutrophils), decreased hemoglobin (74.0 g/L), elevated urea (13.70 mmol/L), creatinine (728 µmol/L), uric acid (341.0 µmol/L), and a reduced estimated glomerular filtration rate (5.7 mL/min/1.73 m²). Procalcitonin was elevated at 4.80 ng/mL, high-sensitivity C-reactive protein at 219.6 mg/L, and erythrocyte sedimentation rate at 117 mm/h. The unclear nature of the abdominal mass and infection complicated the decision-making process. After a multidisciplinary consultation with a radiologist, infectious disease specialist, general surgeon, and gynecologist, an abdominal Magnetic Resonance Imaging (MRI) with Diffusion Weighted Imaging (DWI) was performed (October 2023). The findings revealed peritoneal thickening with restricted diffusion and pelvic effusion, suggesting a potential infectious lesion. A pelvic mass with indeterminate signals on CT raised the suspicion of ovarian teratoma (Figs. 1A and 1B). The consensus was an intra-abdominal abscess, although the possibility of a teratoma could not be ruled out. Fever precluded immediate abdominal surgery. Therefore, ultrasound-guided aspiration and drainage were performed at two sites with the highest collection of pelvic abscesses. Subsequently, Microbial cultures from the drainage fluid revealed MDR *Escherichia coli* sensitive to avibactam, leading to daily intravenous and intraperitoneal avibactam therapy. In this period, yellow-green purulent secretions were drained daily (Fig. 1C), along with fat-like substances (Fig. 1D). From October 21st to 29th, the patient’s body temperature remained stable at approximately 36.8 °C without recurrent fever. Gradual decline in serum WBC and PD fluid WBC following drainage and targeted antibiotics (Supplementary Fig. 2). On October 30th, a hair-like structure (Fig. 1E) was flushed out during peritoneal irrigation. Based on these findings, a multidisciplinary consultation including obstetrics/gynecology oncology, radiology, gastrointestinal surgery, and anesthesiology concluded the mass was likely a teratoma with abscess formation. They advised surgical excision post-fever control. However, on October 30th, the patient's body temperature rose again to 38.0°C. According to the suggestions from microbiology experts, the antibiotic medicine was changed to avibactam sodium plus aztreonam. The patient’s temperature subsequently stabilized, and the obstetrics and gynecology team, along with the gastrointestinal surgery team, performed the mass resection. Postoperative pathology confirmed the presence of mature teratoma (Fig. 1F). Afterwards, the patient continued with maintenance hemodialysis and did not experience fever again. Normalization of serum WBC and PD fluid WBC after teratoma excision (Supplementary Fig. 2). A post-excision CT (November 2023) showed resolution of the pelvic mass but increased residual gas-fluid levels, consistent with postoperative changes.

## Discussion

Peritonitis, a prevalent complication in peritoneal dialysis (PD), has an annual incidence in China of 0.7–1.0 episodes per 100 patient-years [4]. While many cases can be effectively managed with standard treatments, the rising incidence of multidrug-resistant infections has become a significant concern [5]. MDR organisms, including Methicillin-resistant *Staphylococcus aureus* (MRSA), Methicillin-resistant *Staphylococcus epidermidis* (MRSE), Carbapenem-Resistant *Klebsiella pneumoniae* (CRKP), and extended-spectrum β-lactamases (ESBLs), can complicate treatment. These resistant organisms form robust biofilms, enabling them to persist in the PD environment and causing infections, necessitating more aggressive and comprehensive antibiotic coverage [1].

Beyond conventional risk factors, the interplay between end-stage renal disease (ESRD) etiology and immune dysfunction may further predispose PD patients to refractory infections. Although the underlying renal pathology in this case remains unconfirmed, epidemiological patterns suggest chronic glomerulonephritis as the most probable etiology—accounting for 40–55 % of ESRD cases in young Chinese adults without diabetes or hypertension [6]. Chronic glomerulonephritis is characterized by persistent systemic inflammation, with elevated cytokines (e.g., IL-6, TNF-α) impairing neutrophil chemotaxis and macrophage phagocytosis [7]. These immunological perturbations, compounded by uremia-induced T-cell anergy and hypogammaglobulinemia [8], create a permissive environment for multidrug-resistant (MDR) pathogen colonization [9]. This bidirectional relationship—where ESRD exacerbates infection susceptibility, and recurrent infections accelerate renal decline—demands dual management strategies.

In the case of this study, diagnosis at an external facility revealed diverse pathogens, including *Gram-positive cocci*, *Gram-negative rods*, *fungi* and multidrug-resistant *Pseudomonas aeruginosa*. An unidentified abdominal mass with abscess complicated management, and fever persisted post-PD catheter removal and abscess drainage. Upon admission to our department, we detected multidrug-resistant *Escherichia coli* in the abdominal cavity, further complicating treatment. Following admission to our department, *P. aeruginosa* was no longer isolated. Based on the patient’s prior clinical course, we identified multidrug-resistant *E. coli* as the primary pathogen. Targeted therapy with the sensitive antibiotic ceftazidime-avibactam led to improved temperature control [10].

The patient’s abdominal mass with multiloculated abscesses posed dual diagnostic and therapeutic challenges. First, the acute presentation with PD-associated peritonitis prioritized urgent abscess drainage over tumor investigation, as inflammatory exudates obscured teratoma features on initial laparoscopy and CT findings overlapped with chronic infection markers, delaying recognition of its neoplastic nature. Antibiotics struggle to penetrate these inflammatory barriers [11], as evidenced by our observation that ceftazidime-avibactam concentrations in cyst fluid remained lower than serum levels. Second, the coexistence of biofilm-protected pathogens (*P. aeruginosa* on catheter tip) and teratoma-derived microbiota (*E. coli* from cyst contents) created a polymicrobial milieu requiring combinatorial strategies. Antibiotic monotherapy may not prevent relapse and carries risks of hepato-renal toxicity or secondary mycoses. Thus, combining antibiotics with drainage or surgical intervention enhances treatment efficacy [12]. Multidisciplinary abscess management via aspiration, drainage, and irrigation stabilized the patient’s temperature. Hair-like material found during irrigation suggested a teratoma, later confirmed by pathology. During later treatment stages, the patient had a transient fever, with *E. coli* maybe as New Delhi metallo-β-lactamase (NDM)-producing. While ceftazidime-avibactam alone is ineffective, combining it with aztreonam offers strong antibacterial effects against NDM [13]. This combination controlled the fever, enabling further surgical intervention.

This case illustrates a transition from catheter-related peritonitis to teratoma-driven refractory infection. Initially, catheter colonization by *P. aeruginosa* and *Enterococcus* likely seeded the peritonitis **(**Supplemental Fig. 1**)**. However, the teratoma’s mechanical and inflammatory effects perpetuated the infection through three mechanisms: 1) Gut microbiota translocation via peritoneal microtears, introducing *E. coli* and *Klebsiella*; 2) Abscess formation adjacent to the teratoma, shielding pathogens from antibiotics; 3) Immune exhaustion from chronic inflammation and malnutrition. While teratomas are rare in PD patients, this case underscores the importance of evaluating for structural comorbidities in refractory peritonitis. Even sterile masses can perpetuate infection through indirect mechanisms, necessitating imaging (e.g., CT/MRI) and multidisciplinary management. Early surgical intervention, as performed here, remains critical to eliminate anatomic niduses of infection.

## Conclusion

This report presents a rare PD-associated infection with abdominal teratoma and abscess. Key clinical insights include: 1) In cases of persistent infection in PD patients, it is essential to identify underlying factors affecting infection control rather than intensifying antibiotic regimens; 2) Continuous drainage and prompt surgery are critical for abscess management and multidrug-resistant organisms (MDROs) prevention; 3) In reproductive-age female PD patients with abdominal masses and abscesses, the possibility of a teratoma should be considered.

## Ethical approval

This study has been approved by the Ethics Committee of Huazhong University of Science and Technology (TJ-IRB20230836), and informed consent was obtained from the patient.

## Consent

Yes, written informed consent was obtained from the patient for their anonymized information to be published in this article.

## Funding

The author(s) received no financial support for the research, authorship, and/or publication of this article.

## CRediT authorship contribution statement

**Mao Liyan:** Writing – review & editing, Formal analysis, Data curation. **Tang Yi:** Formal analysis, Data curation. **Yang Juan:** Writing – review & editing, Writing – original draft, Supervision, Methodology, Investigation.

## Supplemental material

Supplemental material for this article is available online.

## Declaration of Competing Interest

The authors declared no potential conflicts of interest with respect to the research, authorship, and publication of this article.

## References

[1] Guo S., Yang L., Zhu X. (2023). Multidrug-resistant organism-peritoneal dialysis-associated peritonitis: clinical and microbiological features and risk factors of treatment failure. Front Med.

[2] Iwahashi N., Deguchi Y., Horiuchi Y. (2018). Live birth following laparoscopic fertility-sparing surgery for papillary thyroid carcinoma arising from mature ovarian cystic teratoma: a case report. Mol Clin Oncol.

[3] Li Philip Kam-Tao, Chow Kai Ming, Cho Yeoungjee (2022). ISPD peritonitis guideline recommendations: 2022 update on prevention and treatment. Perit Dial Int.

[4] Yu X., Yang X. (2015). Peritoneal dialysis in China: meeting the challenge of chronic kidney failure. Am J Kidney Dis.

[5] Li P.K., Chow K.M., Van de Luijtgaarden M.W. (2017). Changes in the worldwide epidemiology of peritoneal dialysis. Nat Rev Nephrol.

[6] Zhang L., Long J., Jiang W. (2016). Trends in chronic kidney disease in China. N Engl J Med.

[7] Lech M., Anders H.J. (2013). The pathogenesis of lupus nephritis. J Am Soc Nephrol.

[8] Aytekin G., Baloğlu İ., Çölkesen F. (2021). Nephrological factors may cause kidney dysfunction in patients with common variable immunodeficiency. Turk J Med Sci.

[9] Arpaia N., Campbell C., Fan X. (2013). Metabolites produced by commensal bacteria promote peripheral regulatory T-cell generation. Nature.

[10] Adamkova V. (2019). The role of new antibiotics in intra-abdominal infections in the era of multi-resistant bacteria. Rozhl Chir.

[11] Mechai F., Kolakowska A., Carbonnelle E. (2023). Intra-abdominal abscesses: microbiological epidemiology and empirical antibiotherapy. Infect Dis Now.

[12] Shanturov V.A., Kogan A.S., Boiko T.N. (2000). Puncture-drainage sanation of abdominal abscesses: is it sufficient treatment?. Khirurgiia.

[13] Falcone M., Daikos G.L., Tiseo G. (2021). Efficacy of ceftazidime-avibactam plus aztreonam in patients with bloodstream infections caused by metallo-beta-lactamase-producing Enterobacterales. Clin Infect Dis.

