# Supplementary material for: A complicated case of refractory multiple drug-resistant peritoneal dialysis-associated peritonitis due to teratoma
Source: IDCases. 2025 Mar 31;40:e02209. doi: 10.1016/j.idcr.2025.e02209 (PMC11999219; doi:10.1016/j.idcr.2025.e02209)
Supplement: Supplementary file 1 — Supplementary material [file mmc1.docx]

**
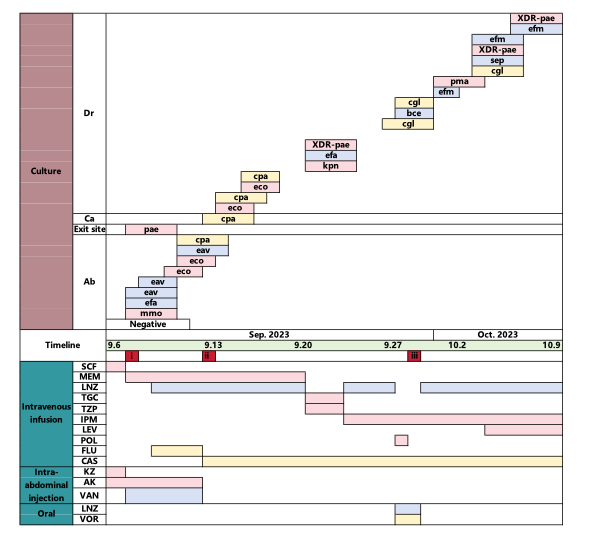
Supplemental Figure 1. The timeline of the patient's diagnostic and therapeutic history at an external facility before admission**. Note: Gram-positive bacteria and corresponding antibiotics are labeled in blue, Gram-negative bacteria and corresponding antibiotics are labeled in pink, and fungi along with antifungal. The Red boxes indicate the presence of clinical symptoms or signs in the patient. i) The patient exhibits abdominal pain and meningeal irritation signs. ii) At this time point, the patient's peritoneal dialysis catheter is removed, and an abdominal drain is placed. iii) The patient discontinues colistin due to ototoxicity. Abbreviation: Dr/drainage, Ca/Catheter, Ab/Abdominal fluid. mmo/Morganella morganii, efa/Enterococcus faecalis, eav/Enterococcus avium, eco/Escherichia coli, cpa/Candida parapsilosis, pae/Pseudomonas aeruginosa, kpn/Klebsiella pneumoniae, cgl/Candida glabrata, bce/Bacillus cereus, efm/Enterococcus faecium, pma/Stenotrophomonas maltophilia, sep/Staphylococcus epidermidis. SCF Cefoperazone/Sulbactam, MEM Meropenem, LNZ Linezolid, TGC Tigecycline, TZP Piperacillin/Tazobactam, IPM Imipenem, LEV Levofloxacin, POL Polymyxin B, FLU Fluconazole, CAS Caspofungin, KZ Cefazolin, AK Amikacin, VAN vancomycin, VOR Voriconazole.

**
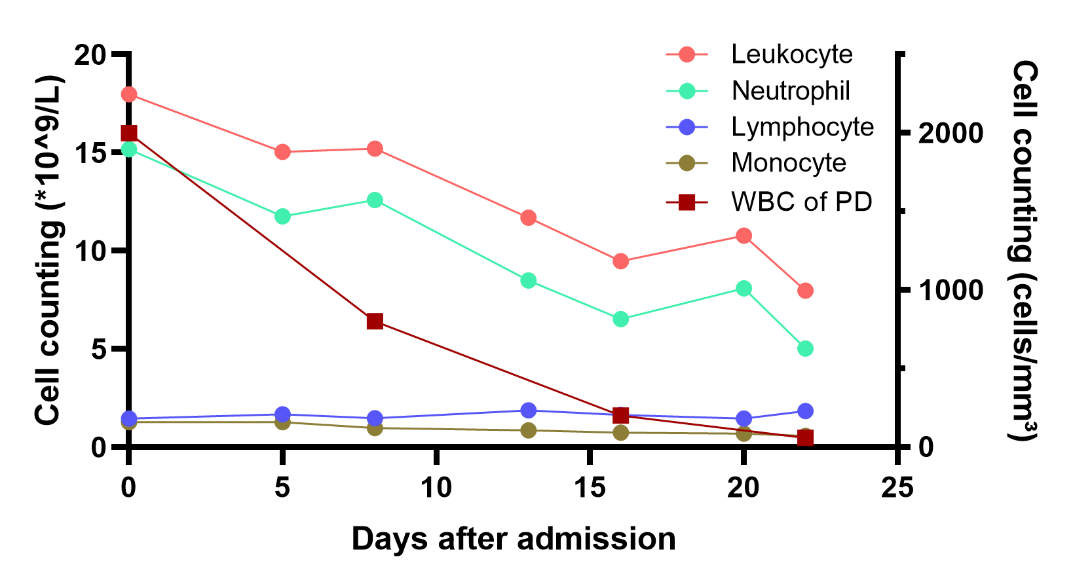
**

**Supplemental Figure 2. Dynamic changes of PD fluid and serum inflammatory indicators during hospitalization.** WBC denotes white blood cell of PD fluid. Leukocyte, Neutrophil, Lymphocyte and Monocyte means the cell counting of serum.
